# Capuchin Red Kite–optimized Swin Transformer-based Convolutional Block Attention Module for Early Diagnosis and Classification of Pneumonia

**DOI:** 10.2174/0115734056392646251021111437

**Published:** 2025-11-28

**Authors:** Shaik Sikindar, Ch V. Raghavendran, G. Madhavi

**Affiliations:** 1 Department of Computer Science and Engineering, Jawaharlal Nehru Technological University Kakinada, Kakinada, Andhra Pradesh, 533003, India; 2 Department of IT, Aditya College of Engineering and Technology, Surampalem, India; 3 Department of Computer Science and Engineering, University College of Engineering Narasaraopet, JNTUK Kakinada, Andhra Pradesh, 522601, India

**Keywords:** Pneumonia, Chest X-ray, CT scan images, Capuchin red kite optimization, Adaptive gated recurrent units, Swin transformer, convolutional block attention module

## Abstract

**Introduction:**

Pneumonia is a serious respiratory disease that requires early and precise diagnosis to reduce morbidity and mortality. This study aims to develop an efficient deep learning model for the accurate classification of pneumonia, COVID-19, and normal cases using chest X-ray and CT images.

**Methods:**

The proposed model combines Capuchin Red Kite Optimization (CRKO) with a Swin Transformer–based Convolutional Block Attention Module (ST-CBAM). A Butterworth filter is applied during preprocessing to enhance image quality. ResNet and Vision Transformer are used for feature extraction, capturing local and global patterns, respectively. These features are fused using Adaptive Gated Recurrent Units (AGRU) and optimized with CRKO. The model is trained and validated using a publicly available chest X-ray dataset from Kaggle.

**Results:**

The model achieved classification accuracies of 99% for normal, 99.9% for COVID-19, and 98.2% for pneumonia cases. It recorded an AUC of 98.93%, outperforming existing models such as ACNN, 3D-CNN, LWHNN, and CA-DCNN in both accuracy and execution time.

**Discussion:**

The integration of CRKO with ST-CBAM, along with hybrid feature extraction and fusion techniques, contributes to the model’s high performance. The results indicate a strong potential for clinical application. However, future studies should validate the model across diverse, real-world datasets to ensure generalizability.

**Conclusion:**

The proposed deep learning framework offers a fast, accurate, and reliable solution for automated pneumonia diagnosis, showing promise for deployment in medical imaging systems.

## 1 INTRODUCTION

Pneumonia is a lung inflammation that mostly affects the alveoli, whichare tinyairsacs. Inthehumanrespiratory system, the lungs are among the most important organs [1]. Pneumonia is frequently caused by viruses or microbes and manifests as coughing, fever, and dyspnea. It is one of the leading respiratory infections contributing to pediatric mortality globally, according to the United Nations Children's Fund. There is a notable disparity in the prevalence of pediatric pneumonia between industrialized and developing nations [2]. If not promptly managed, childhood pneumonia can result in difficulty breathing and can be fatal. Nevertheless, early detection and treatment can cure the majority of cases.

Pneumonia is typically caused by bacterial or viral infections, though other microbes are less frequently responsible. Determining the specific pathogen can be challenging. Medical evaluation and clinical signs are frequently used to make a diagnosis. The diagnosis may be confirmed by sputum culture, blood testing, and chest X-rays [3]. The most prevalent causes of pneumonia are viral or microbial infections, with fungi and parasites being less frequent. Although over 100 strains have been discovered, only a small number account for the majority of infections. About 15% of illnesses in adults and 45% of illnesses in children can be associated with both viruses and bacteria [4]. Physicians can assess the severity of respiratory infections using chest X-ray images to identify lung lesions [5].

Several issues arise when diagnosing pediatric pneumonia based solely on radiologists’ evaluation. First, there is a significant disparity between the number of physicians and patients in developing countries with a high prevalence of pediatric pneumonia. Second, the expertise and attentiveness of radiologists significantly impact diagnostic reliability. Lastly, radiologists face high workloads due to the growing number of cases, and fatigue may reduce diagnostic accuracy. Therefore, the adoption of automated diagnostic technologies is essential to assist radiologists in reducing their workload and accelerating diagnosis.

Pneumonia can be classified in several ways: by mode of transmission (community-acquired, healthcare-associated, hospital-acquired, ventilator-associated) [6]; by causal pathogen; or by the affected lung region (lobar pneumonia, bronchial pneumonia, interstitial pneumonia). Pediatric pneumonia can also be categorized as non-severe, severe, or very severe based on clinical manifestations [7]. The environment in which pneumonia arises is significant for treatment because it influences the likely pathogens, the expected effective medications, and the anticipated outcomes based on the patient’s condition. Certain forms of pneumonia can be prevented with vaccination. Additional preventive strategies include maintaining good hygiene, avoiding smoking, and strengthening the immune system through regular exercise and a nutritious diet. To help ease breathing, moisturizing agents, warm showers, and warm liquids can be beneficial [8].

The most common complications of pneumonia include respiratory failure, septicemia, organ dysfunction, bleeding disorders, and exacerbation of pre-existing medical conditions. Early diagnosis can reduce both mortality and morbidity, and the overuse of broad-spectrum antibiotics should be avoided.

This work presents a novel Capuchin Red Kite–Optimized Swin Transformer–based Convolutional Block Attention Module for the early diagnosis and classification of pneumonia. The following points summarize the contributions of the proposed study.

- **Reducing Noise:** From the chest X-rays or CT scans, noisy data is removed during preprocessing using a Butterworth filter. This is followed by feature extraction, resulting in cleaner and more suitable images.
- **Enhancing Image Quality:** Relevant lung features are highlighted using the Butterworth filter, improving the accuracy of diagnostic classification.
- **Feature Capturing:** Both local and global features of lung images are extracted *via* ResNet and Vision Transformer models.
- **Feature Fusion:** Features from various sources are fused using the AGRU model. More relevant features are selected to provide optimal diagnostic performance.
- **Detection and Classification:** The three categories, normal, COVID-19, and pneumonia, are classified using the CRKO-optimized ST-CBAM model.

To address the diagnostic complexity of pneumonia from chest X-ray images, the proposed approach integrates multiple specialized modules, each designed to solve a specific challenge. Adaptive Gated Recurrent Units (AGRU) are used to dynamically fuse local and global features extracted from ResNet and Vision Transformer, capturing fine-grained details and contextual patterns in the lung region. The Swin Transformer with Convolutional Block Attention Module (ST-CBAM) provides both hierarchical spatial feature representation and fine-level attention to lung abnormalities. Furthermore, Capuchin Red Kite Optimization (CRKO) is used to automatically optimize network hyperparameters and attention configurations, ensuring efficient convergence and improved classification performance. This modular integration forms a synergistic architecture tailored for robust, scalable, and interpretable pneumonia diagnosis.

This section outlines the structure of the rest of the paper: Section 2 summarizes pneumonia prediction along with a review of existing works. Section 3 presents the design of the proposed approach, and Section 4 discusses the experimental investigations. The paper concludes with Section 5.

## 2 RELATED WORKS

Numerous convolutional neural network (CNN)-based recognition techniques for COVID-19 chest X-ray images have been presented. Feng *et al*. [9] described an attention-based CNN (ACNN), designated as PCXRNet, for the analysis of chest X-ray images in pneumonia classification. A condensed attention module (CDSE) consists of two phases: the squeeze-excitation phase and the condensation phase, to optimize the efficiency of information extraction from the channels of the feature maps. A multi-convolution spatial attention module (MCSA) helps the framework focus more on relevant spatial elements in each feature map. The proposed network achieved a precision of 94.619% in diagnosing COVID-19. However, subtype identification was not included. Early detection of COVID-19 is crucial for both protecting the unaffected population and providing patients with appropriate healthcare. The network dynamically distinguished COVID-19 from community-acquired pneumonia (CAP) in chest CT using a dual-sampling attention network.

Ouyang *et al*. [10] presented an online attention module with a 3D CNN that focuses on the infected areas of the lungs during diagnosis. A dual-sampling technique was created to address class imbalance. Particularly in the early stages of the COVID-19 pandemic, this approach helped radiologists diagnose COVID-19 from CAP. However, the precision for small-infection-area COVID-19 cases was not sufficient. Quick detection of COVID-19 patients is essential to limit viral spread and avoid hospital overcrowding.

Several networks have been developed to assist radiologists in diagnosing COVID-19 using CT scans. The COVID-CT dataset includes 324 positive patient cases from three publicly available sources. Li *et al*. [11] developed a lightweight hybrid neural network (LW-HNN) incorporating a Convolution Block Attention Module to learn potent visual feature representations while reducing parameters. This approach improved the effectiveness and precision of COVID-19 diagnosis, aiding in virus containment, though the system lacked processing capacity.

Chest X-ray remains the most widely used non-invasive and painless diagnostic procedure for pediatric pneumonia. However, due to low radiation levels, accurate detection can be challenging, necessitating reliable computer-aided diagnosis (CAD) systems. Asswin *et al*. [12] implemented stacking ensemble learning on features extracted from channel attention–deep CNN structures (CA-DCNN). For final normal *versus* pneumonia classification, gradient boosting, logistic regression, a stacking classifier with support vector machine, and K-nearest neighbors were used. The method achieved 96.15% reliability for real-time pediatric pneumonia detection. However, this procedure is time-consuming and heavily depends on expert availability. Due to the similarity of pneumonia lesions, manual recognition can be misinterpreted and slow.

Wong *et al*. [13] developed a multi-scale attention network (MSANet) that leverages multiple state-of-the-art deep learning methods to automatically classify COVID-19 and various pneumonia forms. The approach considers multi-scale features of pneumonia lesions and discriminative information, achieving 97.46% accuracy across multiple pneumonia classes. However, large-scale CT data collection remained insufficient. The framework can effectively identify pneumonia caused by COVID-19 and other viruses inducing interstitial lung disease (ILD), while also predicting the likelihood of pneumonia presence in a CT scan.

Wang *et al*. [14] proposed a multi-task prior-attention learning strategy using volumetric chest CT scans for COVID-19 diagnosis. A prior-attention residual learning block combined two ResNet-based branches into a single model for end-to-end training. These blocks allowed the COVID-19 classification branch to develop stronger discriminative features by transferring hierarchical attention information from the lesion segmentation branch. Additional supervision data with precise lesion locations improved classification efficiency. However, obtaining sufficient samples simultaneously can be challenging.

Chest X-ray remains the primary diagnostic method for most respiratory illnesses. Malik *et al*. [15] demonstrated a multi-class deep learning approach recognizing TB, pneumonia, COVID-19, and lung cancer (LC) from chest X-rays. CDC-Net, a CNN model comprising dilated convolutions and residual connections, effectively identified COVID-19 and other chest conditions. However, it does not remove irrelevant data in feature maps. Large multicenter chest X-ray (CXR) datasets enable deep neural networks (DNNs) to perform disease recognition, lesion segmentation, and report generation efficiently.

Unsupervised domain adaptation (UDA) techniques help reduce cross-domain discrepancies. Li *et al*. [16] proposed an attention-guided partial domain adaptation (AGPDA) network to address negative transfer issues. It consists of two modules: a lightweight region-wise multi-adversarial network implementing a region attention discrimination block to produce fine-grained attention, and a residual feature recalibration block with class-weighted maximum mean discrepancy loss to down-weight irrelevant source features. This method improved efficiency with an accuracy gain of 4.35%, though overfitting issues were not fully addressed.

Building on prior CNN and attention-based methods for COVID-19 and pneumonia diagnosis, Liu *et al*. [17] demonstrated that Vision-Language Models (VLMs) offer a more generalizable and data-efficient approach by integrating visual features with textual semantics. Unlike traditional single-modality models, VLMs enable cross-modal reasoning and support zero-shot and few-shot tasks, addressing challenges like label scarcity and domain adaptation. Applications extend beyond classification to report generation, question answering, and segmentation, advancing AI-driven medical diagnostics.

## 3 MATERIALS AND METHODS

Pneumonia presents varying severity, and early and accurate detection from medical images is critical. This section provides a step-by-step explanation of the proposed multi-module deep learning pipeline tailored for pneumonia classification. The proposed architecture (Fig. **1**) addresses three core challenges: (1) how to effectively extract and combine both local and global features from chest X-ray images, (2) how to enhance attention to spatially and channel-wise relevant areas in the lungs, and (3) how to optimally tune deep learning parameters for accurate and generalizable classification. These challenges are addressed, respectively, by the use of Adaptive GRU-based fusion, Swin Transformer with CBAM, and Capuchin Red Kite Optimization (CRKO).

### 3.1 Butterworth Filter

The input X-ray images often contain noise and are of low quality due to fluids or congestion affecting the lung regions. To ensure model generalizability and enhance image quality across the dataset, a Butterworth filter was applied, along with image resizing and normalization. Pixel intensity values were adjusted to a consistent range through normalization. High-frequency noise was smoothed using a low-pass filter, which removes unwanted artifacts [18]. The images were resized, and frequency components were controlled to preserve critical features. These preprocessing steps enhance robustness to variations in the dataset and improve model generalizability.

### 3.2 Feature Extraction

A critical step is feature extraction, which enables accurate detection and classification of normal, COVID-19, and pneumonia cases through effective feature representation. From the preprocessed output, key information is extracted and identified using both local and global feature extraction procedures.

#### 3.2.1 ResNet for Extracting Local Features

ResNet is used to extract local features, leveraging its capacity to capture deep hierarchical features along with residual learning. This is accomplished by optimizing residual mapping using feed-forward neural networks [19]. Identity mappings are added as additional layers to mitigate training error. Conventionally, layers are stacked directly to achieve the desired mapping. Here, the residual mapping is attained using the layered stack. Consider that the mapping to be obtained is designed to match the various nonlinear layers and can be expressed as Eqs. (**1** and **2**).

**Table:** 

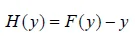	(1)

**Table:** 

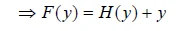	(2)

The skipping of layers is implemented using shortcut connections, such as vector addition for the identity function during backpropagation, which supports residual learning. No additional parameters are required for the identity mapping. Edges and textures are extracted from each layer of the ResNet identity mapping. This provides improved local features that are used for the diagnosis and classification of pneumonia.

#### 3.2.2 Vision Transformer (VT) for Global Feature Extraction

VT is a third-stream model of CNN and is designed based on the attention mechanism, correlating pixels that are both nearby and far apart. This provides detailed information about the contextual relationships in the lung area [20]. The images are first split into small patches to perform the attention mechanism. Hence the image that is represented in *I* ϵ *R^hxwxc^* which is converted into *P* ϵ *R^Mxps^2^xc^* . Here, the width and height of the images are implied with w and h. The number of channels is indicated as c with the M number of patches and evaluated as Eq. (**3**),

**Table:** 

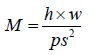	(3)

The patch P and the size is ps. After splitting the patches, the raw image I is transformed into 2D matrix P with the linear projection of dimensionality 64 as given *via* Eqs. (**4**, **5**),

**Table:** 

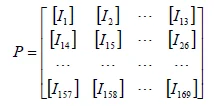	(4)

**Table:** 

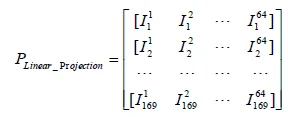	(5)

Positional embedding is performed to group the image patches into smaller groups for the implementation of larger image sizes. This is effectuated to reduce the computational expense of the transformer. The position embedding is performed with the merging of frequencies of sine and cosine functions. The odd position patches are embedded using the cosine function and the sine function for the even position is used. The position is implied using the parameter PO and the dimensionality is indicated with d. The maximum length of the patch group is designed as l. The embedded patch is generated with the concatenation of the linear projected patch with the positional embedding and is implied using given Eqs. (**6, 7**),

**Table:** 

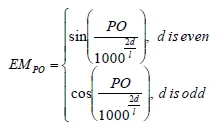	(6)

**Table:** 

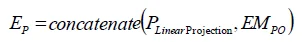	(7)

The embedded patch, obtained from linear projection and positional embedding, is passed to the encoder block. The encoder consists of six layers: a normalization layer, a multi-head self-attention layer (MHSAL), and a multilayer perceptron (MLP). The input is concatenated with the MHSAL layer’s output at the encoder’s input block [21]. The processed or extracted features are then forwarded to the normalization layer, and finally, the output passes through a dropout layer in the MLP. Positional information and its effect are enhanced by adding the attention output from the skip connection, which improves the original embedded patch. The attention is computed using the embedding metrics: key (k), query (q), and value (v). The respective weight matrices are denoted by the parameters *W_k_*, *W_q_* and *W_v_* and can be expressed *via* Eqs. (**8**-**10**).

**Table:** 

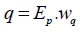	(8)

**Table:** 

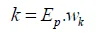	(9)

**Table:** 

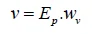	(10)

Where, *w^q^*, *w^k^*, *w_v_* ϵ *R^l_model_^x^l_k_^* with the embedded patch. The contextual relationship between the two image patches and the scoring function is based on the attention value *via* Eqs. (**11, 12**).

**Table:** 

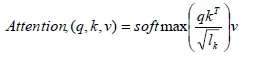	(11)

**Table:** 

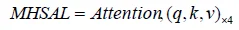	(12)

The dense layer with the MLP and the activation layer of Gaussian error linear unit (GELU) are in Eq. (**13**) for the later layers. The output layer can be described *Trasformer_Feature_Shape_* with the cumulative distribution of the Gaussian distribution (*θ*) as Eqs. (**14, 15**),

**Table:** 

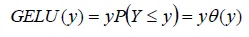	(13)

**Table:** 

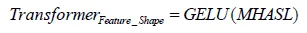	(14)

**Table:** 

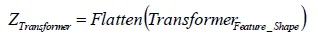	(15)

The features that are extracted from both blocks is forwarded to the fusion block.

### 3.3 Adaptive GRU for Feature Fusión

The proposed approach adopts an innovative technique for the fusion of features from ResNet (edges and textures) and VT (contextual relationships between pixels). This method skillfully integrates the inputs provided to the block in a dynamic manner. The schematic model of the Adaptive GRU for feature fusion is illustrated in Fig. (**2**). Two learnable weight parameters, α and β, are included to regulate the system. This allows the model to capture complex temporal correlations and enhances pneumonia prediction accuracy [22]. The input is a tensor *X*, where the length of the input sequence is *L* and *d* is the dimension. The input *X* is forwarded to a multilayer GRU, producing a feature vector *F*. The number of hidden units is denoted as *g*. The new fused segmented sequence, provided with the corresponding patch, is denoted as *S*, where the length of the segment is *P* with a total of *M* patches. The processing of *S* is performed with the multilayer GRU, resulting in the feature vector. *F'* The novel sequence obtained is treated as *S'* and forwarded to the multilayer GRU, producing the outcome *F''*. The finalized feature representation is obtained using the weighted sum of the learnable parameters α and β along with *F'* and *F''*. This fused feature is then passed to the prediction block to obtain the predicted output. The schematic model of the Adaptive GRU for feature fusion is shown in Fig. (**2**).

To integrate local and global information, a weighted summation fusion is applied, defined as

**Table:** 

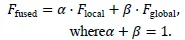

Here, and are learnable fusion weights, optimized during training to prioritize the most relevant representations for each sample. The resulting fused feature vector is then passed through a linear head for downstream classification *via* the ST-CBAM block.

### 3.4 Detection and Classification

The early detection and classification of pneumonia are achieved using the integration of Capuchin Red Kite Optimization (CRKO) with the Swin Transformer-based Convolutional Block Attention Module (ST-CBAM). The multi-class predictions of normal, COVID-19, and pneumonia cases are predicted and categorized with CRKO and ST-CBAM, in which each description of these methodologies is explained in the following section. To optimize the classification task for normal, COVID-19, and pneumonia cases, the categorical cross-entropy loss function was used. This function is appropriate for multi-class classification problems where each sample belongs to one of several mutually exclusive classes. The loss is defined as , where C is the number of classes, is the ground truth label for class iii (encoded as a one-hot vector), and is the predicted probability for class iii from the softmax output. This function penalizes incorrect predictions more heavily when the confidence in the wrong class is high, thereby guiding the model toward more accurate classifications. In this work, the cross-entropy loss was minimized using the Adam optimizer during training.

#### 3.4.1 Swin Transformer

The shifted window attention and hierarchical feature maps are the two major key concepts. Based on multifaceted computer vision tasks, the promising tool is made with advancements to manage large-scale image data efficiently. Various feature levels are represented with the hierarchical feature maps utilized by Swin Transformer [23]. The input data allows for improved comprehension and context understanding. The variable-scale features are captured more effectively, and each block's interaction field is expanded with the shifted window attention mechanism.

The modules of multi-head self-attention (*MSA*) have two consecutive, shifted window-based MSA(*swMSA*) and window-based MSA(*wMSA*). Use layer norm (ln) before every module of these MSAs. The Swin transformer performs more efficiently *via* configuration modules. The number of tokens with the quadratic computational difficulties has (*MSA*) been defined *via* Eqs. (**16, 17**)

**Table:** 

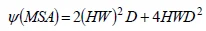	(16)

**Table:** 

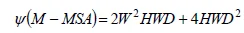	(17)

Where, *HW* is the patch number related to the quadratic relationship exhibited, with D and M, which are constants. The scalable, window-oriented self-attention provides higher values. To compute the non-overlapping windows, cross-window connections are introduced to utilize overlapping windows.

The relationships between positions are captured, and it integrates relative positional bias along with the mechanism of self-attention defined as Eq. (**18**).

**Table:** 

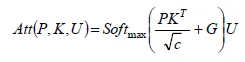	(18)

From this *K*, *P* we *U* are the matrix values of key, query, and value of size. The number of window patches is G.

#### 3.4.2 Convolutional Block Attention Module

The refinement applies CBAM in the proposed framework and is incorporated with the spatial and channel attention modules. The channel attention module receives the input of fused features, which are squeezed along the spatial dimension. Simultaneously, average and max pooling are applied to collect each channel feature map. Simultaneously, max pooling is applied with finer channel attention, and average pooling is employed [24]. One hidden layer is present in the shared MLP, which passes the feature maps through an activation function, ReLU, in the shared MLP. The element-wise summation merges the output feature vectors. The refined feature map is obtained based on channel-wise attention, and the original feature map with channel attention is used to perform attention map-based element-wise multiplication. The following equation computes the attention based on the channel.

**Table:** 

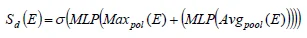	(19)

**Table:** 

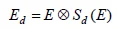	(20)

The channel refined features, input feature, and sigmoid function are in Eqs. (**19**, **20**). The input to the spatial attention module is computed from the channel attention to obtain the channel-refined feature map. To pass through the spatial attention module, the features are concatenated along the channel axis, and average and max pooling operations are applied. The information-rich regions are highlighted effectively by the pooling operations along the channel axis. The input feature of the module, through element-wise multiplication, obtains the final refined feature map *via* Eq. (**21**).

**Table:** 

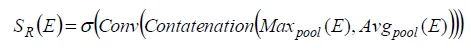	(21)

**Table:** 

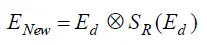	(22)

The attention map *E_New_* in Eq. (**22**) was generated to apply the convolution and pooling that implement the simple attention module present in CBAM.

#### 3.4.3 Swin Transformer-based CBAM

The architectural diagram of the ST-CBAM model is depicted in Fig. (**3**). In CBAM, the channel- and spatial-based attention schemes are combined with the global context modelling of the Swin Transformer for the detection of pneumonia classes. Both global and local contexts are effectively maintained within the shifted windows, which perform multi-head self-attention. The non-overlapping patches in an image are processed with the hierarchical Vision Transformer called ST. Both spatial and channel attentions are sequential modules in CBAM, and significant spatial regions are focused. According to CBAM, the attention mechanisms and the Swin Transformer generate feature map enhancements. The ST receives the fused input image features, and the last stage of the ST receives the feature map, which is then passed to CBAM with its channel attention modules [25]. The weights of channel-based attention are calculated, and the feature maps are multiplied by these weights. Based on the modules of spatial attention, the feature maps are passed, and the spatial attention weights are computed. The feature maps are multiplied, and for downstream tasks, the improved feature maps of CBAM are utilized. The features are hierarchically enhanced using ST along multiple stages by applying CBAM, which demonstrates the detection process in terms of pneumonia detection. Nevertheless, the detection performance of pneumonia classes is enhanced using the Capuchin Red Kite Optimization algorithm.

#### 3.4.4 Capuchin Red Kite Optimization

Here, the Capuchin Red Kite Optimization (CRKO) is used to provide the best solution. The social life simulates a new meta-heuristic model called RKO. For hunting, the lakes, regions, and nests near woods are generated for red kites [26]. To stop trapping in a local optimum, the problem search space is navigated with the meta-heuristic model to provide better outcomes. From the final iterations, an optimum performance is obtained after moving slowly from the stages of exploration toward exploitation. The following eq. (**23**) arbitrarily initializes the position of RK at the primary birds’ positions.

**Table:** 

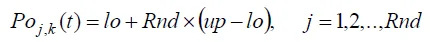	(23)

At iteration *t*, the *^jth^* RK position is *Po_j,k_*(*t*). The boundaries of upper and lower limits are *up* and *lo*. The dimension of the problem and population size are *Dim *and N. From zero to one, the random number implied as *Rnd*..

Eq. (**24**) exemplifies the leadership selection stage and describes the leader selection procedure.

**Table:** 

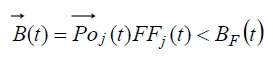	(24)

At iteration t, the optimal best RK position 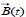 is, and the ^jth^ RK position is indicated 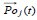. The best bird estimation function value is implied as *B_F_*(*t*).

From the stages of exploration to exploitation, the slow movement required by RK in the case of birds' movements.

**Table:** 

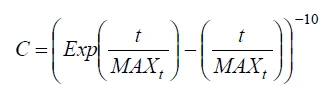	(25)

The maximal iteration and present iterations are described in terms of *MAX_t_* and *t* *via* Eq. (**25**). Following Eq. (**26**), upgrading the position of RK.

**Table:** 

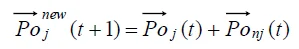	(26)

The new RK position is described 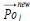 (*t*+1) *via* Eq. (**27**), which is the roulette wheel iteration 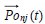. Use the monkey's jump angle (*θ*) of the capuchin search model to upgrade the RK position defined in Eq. (**28**).

**Table:** 

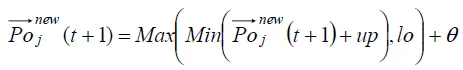	(27)

Where,

**Table:** 

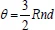	(28)

According to [0, 1] interval, the random number is regularly distributed *Rnd*. Enhance the estimation function to exchange the temporary position. Where, *A_j_new*(*t*+1) is similar to the *Po_j_*(*t*+1).

**Table:** 

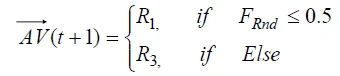	(29)

**Table:** 

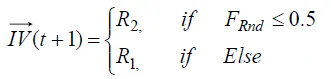	(30)

From this, individual and social arbitrary vectors are 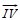 and 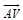 defined in Eqs. (**29** and **30**). The arbitrary vector of *R_1_*, *R_2_* and *R_3_* ranges are [1, 2], [1, 3], and [0, 1]. Figure (**4**) describes the flowchart representation for the CRKO algorithm.

#### 3.4.5 CRKO Algorithm with Swin Transformer-based CBAM

To enhance the detection of pneumonia classes, we propose a unified architecture that integrates the Capuchin Red Kite Optimization (CRKO) algorithm with the Swin Transformer-based CBAM. The AGRU-fused features, which combine local texture (ResNet) and global context (Vision Transformer), are refined through ST-CBAM to focus on spatially significant and diagnostically relevant regions. CRKO then performs metaheuristic optimization of critical model parameters, such as attention kernel sizes, transformer depth, and learning rate, across ST-CBAM layers. This ensures optimal performance through automated tuning, improved convergence, and robustness against data variation. To focus on the channels and informative regions, the fused features are enhanced using CBAM and the shifted window attention mechanism, which provides multi-scale hierarchical feature extraction. The hyperparameter tuning of the CRKO algorithm is then initialized. The solution spaces of exploitation and exploration effectively design the CRKO algorithm. The Swin Transformer-CBAM architecture, including network layer configurations, attention module parameters, and learning rates, is optimized using the CRKO algorithm. For pneumonia disease classification, the most discriminative features are focused on using the CBAM-enhanced output along with the CRKO algorithm [27-31]. Based on ambiguous features, the CRKO algorithm has better search ability and convergence, providing the best solution that tunes the parameters of ST-CBAM. Hence, the CRKO algorithm with ST-CBAM effectively classifies the classes as normal, pneumonia, and COVID-19. The steps are incorporated to predict and classify the pneumonia classes.

- Define the objective function as the performance evaluation of detection, along with accuracy.
- Update the parameters like attention kernel size, reduction ratio, depth, window size, batch size, and learning rate that need to be optimized.
- From CBAM-enhanced outputs, most discriminative features are identified using RKO, and the classification accuracy is enhanced, as well as redundancy is reduced.
- Set the repetition process to evaluate the optimal parameters to enhance the performance of ST-CBAM.
- Detect and classify the normal, pneumonia, and COVID-19 classes and provide minimum error performance with superior accuracy.

### 3.5 Implementation Details

The proposed Capuchin Red Kite Optimized Swin Transformer-based CBAM model was implemented in Python 3.8 using both the TensorFlow 2.10 and PyTorch 1.13 frameworks. Data preprocessing and analysis were performed using standard Python libraries, such as NumPy, Pandas, OpenCV, and Scikit-learn.

All experiments were conducted on a high-performance computing system equipped with an NVIDIA RTX 3090 GPU (24 GB VRAM), Intel Core i9-11900K CPU, 64 GB RAM, and running on Ubuntu 20.04 LTS. The GPU acceleration significantly reduced training time and enhanced model performance, particularly during feature extraction, fusion, and optimization stages.

## 4 RESULTS AND DISCUSSIONS

The experimental evaluation of the proposed model was conducted using a publicly available chest X-ray dataset obtained from Kaggle: https://www.kaggle.com/datasets/pra shant268/chest-xray-covid19-pneumonia. This dataset consists of a total of 6,432 labeled chest X-ray (CXR) images, covering three classes: Normal, Pneumonia, and COVID-19. The dataset includes:

- 1,583 images labeled as Normal
- 576 images labeled as Pneumonia
- 4,273 images labeled as COVID-19

All images were collected from various hospital sources and annotated by radiologists. The images vary in size and resolution, and are provided in PNG/JPEG formats.

Prior to training, all images were pre-processed using a Butterworth filter, resized to a uniform input size, and normalized.

For evaluation, the dataset was randomly split into:

- Training set: 5,144 images (80%)
- Testing set: 1,288 images (20%)

Stratified random sampling was used to preserve the class distribution in both the training and test sets.

Table **1** provides a summary of the dataset characteristics. In addition to the training and testing split, a validation set was derived from the training data to monitor model performance during training and prevent overfitting. Specifically, 10% of the training data (*i.e*., 515 images) was allocated as a validation set, leaving 4,629 images for actual training. This results in a final partition of 4,629 training images, 515 validation images, and 1,288 testing images, maintaining class distribution consistency throughout all subsets.

Figure (**5**) demonstrates the sample images of each category, such as normal, COVID-19, and pneumonia.

### 4.1 Evaluating the Performance

The training and validation of the proposed approach over the dataset were analyzed to fix the biased coefficient and weight factor. The training accuracy is always higher since the proposed system is trained using the labeled dataset. The validation accuracy is lower than the training accuracy, as shown in Fig. (**6**). The training accuracy at the 100th epoch is 0.96, and the validation accuracy is 0.95.

To evaluate the proposed model's learning behavior, both training and validation loss values were monitored across 100 epochs. The validation loss was computed using a separate validation set without updating model parameters, ensuring proper evaluation. Figure (**7**) illustrates the decrease of both training and validation losses over time, demonstrating effective learning and generalization by the model.

To evaluate the model’s discriminative performance across various thresholds, ROC curves and AUC scores were computed for each class: Normal, COVID-19, and Pneumonia. Figure (**8**) presents ROC curves generated using a dense set of threshold values, resulting in multiple turning points, as expected for large-scale validation data. This representation provides a more accurate reflection of the model's classification behavior. The proposed method achieves an AUC of 98.93%, outperforming CA-DCNN (97.18%), LWHNN (95.34%), 3D-CNN (93.84%), and ACNN (91.34%).

Figure (**9**) shows the confusion matrix of the proposed work based on the dataset. It summarizes the prediction outcomes. The normal class predicted correctly includes 1,253 instances, and the wrongly predicted instances total 17, with two instances predicted as COVID-19 and 15 as pneumonia. Meanwhile, the COVID-19 instances correctly predicted a total of 458, with one instance wrongly predicted as pneumonia. The pneumonia instances predicted correctly total 1,330, with 14 instances predicted incorrectly—1 as COVID-19 and 13 as normal.

Table **2** discusses the comparative analysis of execution time. Similar operations, regarding the classification output applied to the underlying model and consistent across each disease class, determine the execution time. Existing methods, including ACNN [9], 3D-CNN [10], LWHNN [11], CA-DCNN [12], and the proposed work, are used for comparison. The proposed work achieves the minimum execution time, with 2.8 s for normal, 3 s for COVID-19, and 2.5 s for pneumonia classes, which is lower than the execution times of ACNN [9], 3D-CNN [10], LWHNN [11], and CA-DCNN [12].

Accuracy is an important parameter for the analysis of pneumonia classification, as shown in Fig. (**10**). The proposed work achieved a model accuracy of 99% for normal instances, 99.9% for COVID-19 classes, and 98.2% for pneumonia classes. This shows that the proposed work effectively classifies an average of 99% of the samples. The higher accuracy reflects the capability of the proposed technique to predict the different classes as normal, COVID-19, and pneumonia. Moreover, the dataset is imbalanced, with more non-pneumonia classes, so it is necessary to analyze various parameters, such as recall, F-score, precision, sensitivity, specificity, kappa, and MCC. From the figure, it is noted that the other techniques, including ACNN [9], 3D-CNN [10], LWHNN [11], and CA-DCNN [12], show lower accuracy.

In the proposed pneumonia classification, the model’s capacity is demonstrated with the F1-score and is illustrated in Fig. (**11**). It provides details about how the model balances the tradeoff between the identification of pneumonia by minimizing the false positives. Moreover, higher details are provided by this model regarding the imbalance in the dataset. The F1-score of the proposed work for normal cases is 98.2%, for COVID-19 cases it provides 98%, and for pneumonia cases, it provides 97.5%. These values are higher than the existing works including ACNN, 3D-CNN, LWHNN, and CA-DCNN, which show lower accuracy.

FNR is otherwise known as the miss rate and is shown in Fig. (**12**). The missed percentage of the proposed work while classifying the normal cases is lower than 1%, at 0.8%. The COVID-19 cases are around 0.5%, and the pneumonia cases are around 1.1%, as shown in the figure. This shows how the model can be used to reduce missed diagnoses. The lower value of FNR shows the effectiveness of the proposed model; however, it is necessary to reduce it further to maintain the FNR and FPR. Meanwhile, the other works, including ACNN, 3D-CNN, LWHNN, and CA-DCNN, show higher FNR, as mentioned in the graphical representation.

The proposed model for the prediction of pneumonia disease is analyzed with FPR, and the FPR should be lower. The FPR of the proposed work for normal cases is 0.8%, for COVID-19 cases 0.1%, and 0.6% for pneumonia cases, as visualized in Fig. (**13**). The FPR must be lower to achieve desirable results, and the proposed method demonstrated this. Meanwhile, the other techniques, including ACNN, 3D-CNN, LWHNN, and CA-DCNN, showed higher FPR, as shown in the figure.

Kappa is an important parameter for the analysis of pneumonia classification and is shown in Fig. (**14**). The proposed work achieved a model Kappa of 97% for normal instances, 98% for COVID-19 classes, and 97.3% for pneumonia classes. This shows that the proposed work effectively classifies an average of 97.5% of the samples. The higher Kappa reflects the capability of the proposed technique to predict different classes: normal, COVID-19, and pneumonia. From the figure, it is noted that other techniques, including ACNN, 3D-CNN, LWHNN, and CA-DCNN, show lower Kappa.

In the proposed pneumonia classification, the model’s performance is evaluated using the Matthews Correlation Coefficient (MCC), as shown in Fig. (**15**). The MCC highlights the model’s ability to balance the trade-off between accurately identifying pneumonia and minimizing false positives. Additionally, the model provides deeper insights into dataset imbalances. Specifically, the MCC achieved by the proposed method is 97.2% for normal cases, 99% for COVID-19 cases, and 97.5% for pneumonia cases. These values surpass those of existing methods, including ACNN, 3D-CNN, LWHNN, and CA-DCNN, which exhibit lower accuracy.

In the proposed pneumonia classification, the model’s performance is evaluated using precision, as illustrated in Fig. (**16**). Precision highlights how well the model balances the trade-off between correctly identifying pneumonia and minimizing false positives. Furthermore, the model provides detailed insights into dataset imbalances. The precision achieved by the proposed method is 98.9% for normal cases, 99.4% for COVID-19 cases, and 98.5% for pneumonia cases. These values are higher than those reported by existing methods, including ACNN, 3D-CNN, LWHNN, and CA-DCNN, which demonstrate lower accuracy.

Recall is a key metric for evaluating pneumonia classification, as shown in Fig. (**17**). The proposed method achieves a recall of 99% for normal cases, 99.2% for COVID-19 cases, and 99.1% for pneumonia cases, demonstrating that, on average, it correctly classifies 99% of the samples. The high recall highlights the model’s effectiveness in accurately predicting all three classes: normal, COVID-19, and pneumonia. In comparison, other techniques, including ACNN, 3D-CNN, LWHNN, and CA-DCNN, exhibit lower recall.

The proposed model for pneumonia prediction is evaluated using specificity, which should be high for effective performance. As shown in Fig. (**18**), the proposed method achieves a specificity of 99% for normal cases, 99.9% for COVID-19 cases, and 99% for pneumonia cases. High specificity indicates the model’s effectiveness in correctly identifying negative cases. In comparison, other techniques, including ACNN, 3D-CNN, LWHNN, and CA-DCNN, demonstrate lower specificity.

### 4.2 Strengths and Limitations

The proposed CRKO with the ST-CBAM model exhibits several strengths. It combines local and global feature extraction through ResNet and Vision Transformer, advanced feature fusion *via* Adaptive GRU, and context-aware attention refinement using CBAM, resulting in high classification accuracy across Normal, COVID-19, and Pneumonia categories. The model also achieves faster inference times compared to other state-of-the-art methods and demonstrates robustness against class imbalance. Moreover, automatic hyperparameter tuning through Capuchin Red Kite Optimization further enhances performance and convergence.

Despite these advantages, the study has certain limitations. The model was trained and validated on a single publicly available dataset, which may limit its generalizability to clinical settings with varied imaging conditions or patient demographics. Additionally, the inclusion of Transformer-based components and optimization algorithms increases computational complexity, potentially necessitating high-end hardware for efficient training. Finally, while the model performs well on static X-ray images, it has not been evaluated on real-time data or other imaging modalities such as CT scans. Future work will aim to address these issues by incorporating diverse datasets, improving computational efficiency, and validating the model in broader clinical environments.

## CONCLUSION

This study proposes a novel deep learning framework that combines Capuchin Red Kite Optimization (CRKO) with a Swin Transformer-based Convolutional Block Attention Module (ST-CBAM) for the early detection and classification of pneumonia, COVID-19, and normal cases from chest X-ray images. The model leverages ResNet and Vision Transformer to extract both local and global features, which are adaptively fused using an Adaptive Gated Recurrent Unit (AGRU). The fused features are further refined through ST-CBAM, while CRKO is employed for hyperparameter optimization to enhance classification performance.

Experimental evaluation was conducted on a publicly available Kaggle chest X-ray dataset comprising 6,432 images. The proposed model achieved high classification accuracy: 99% for normal, 99.9% for COVID-19, and 98.2% for pneumonia cases. It also demonstrated a high AUC of 98.93% and significantly outperformed existing methods, including ACNN, 3D-CNN, LWHNN, and CA-DCNN, in both accuracy and execution time. Moreover, the model achieved low false positive and false negative rates, with F1-scores exceeding 97% across all classes.

These results suggest that the CRKO-ST-CBAM framework provides an effective, fast, and robust solution for pneumonia diagnosis. Future work will focus on expanding dataset diversity, improving computational efficiency, and validating the model in real-world clinical settings.

## Figures and Tables

**Fig. (1):**
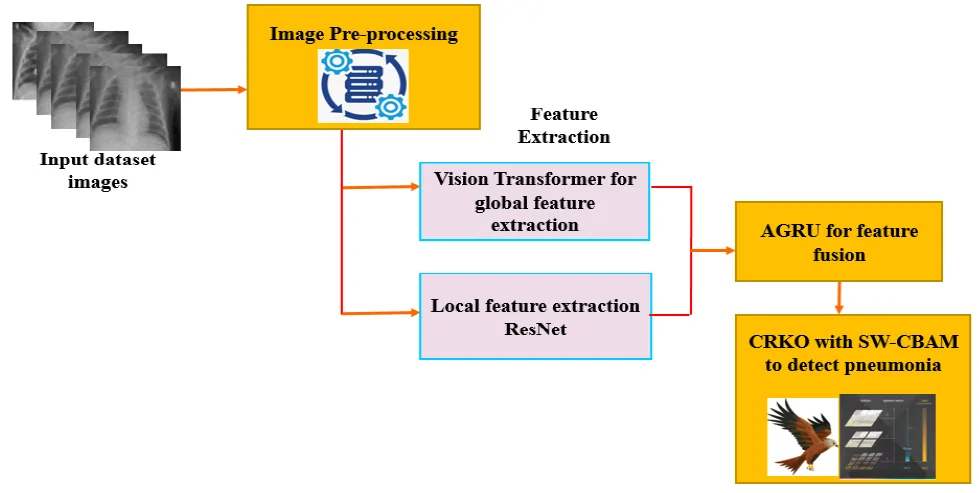
The proposed workflow structure.

**Fig. (2):**
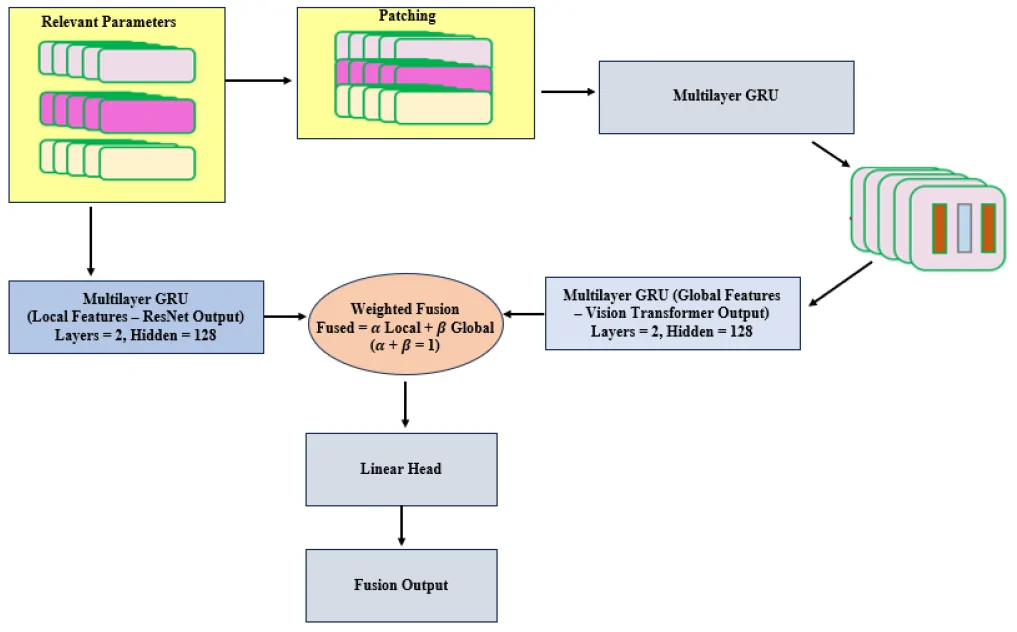
Adaptive GRU-based feature fusion architecture. Local features extracted from ResNet and global features obtained from the Vision Transformer are processed through separate multi-layer GRUs. Each GRU stream consists of two stacked GRU layers, with 128 hidden units per layer, to capture sequential and contextual dependencies within their respective feature spaces. The output of each GRU is represented as a feature vector of shape (*B* x *T* x *d*) where *B* denotes the batch size, *T* the time steps (corresponding to the number of patches or regions), and *d* the hidden size.

**Fig. (3):**
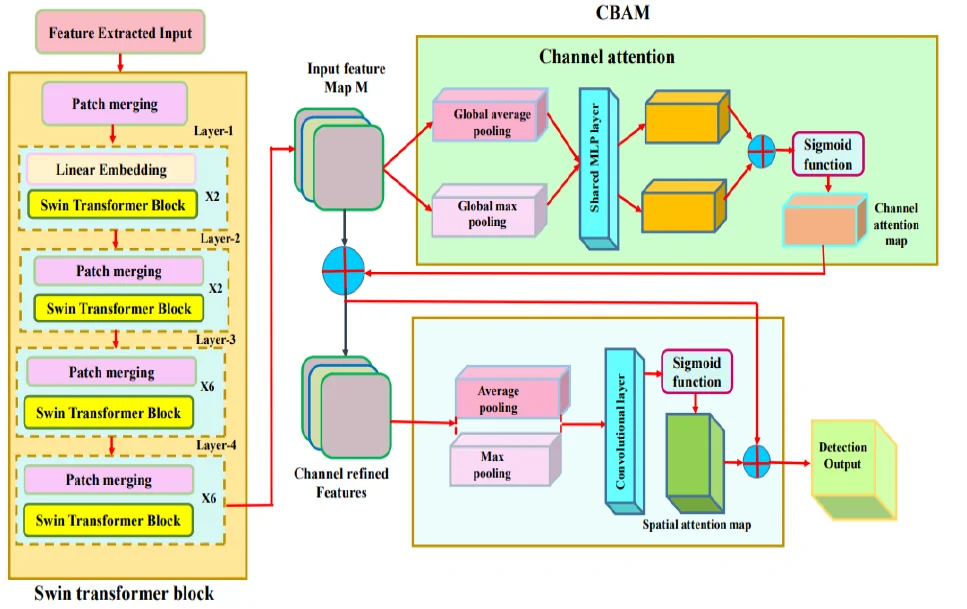
Architecture of ST-CBAM model.

**Fig. (4):**
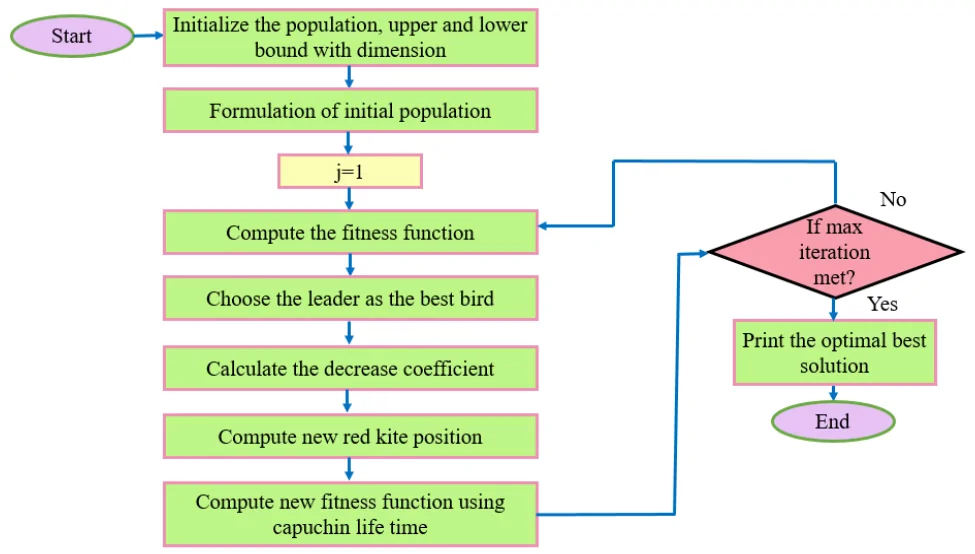
The flowchart representation based on the CRKO algorithm.

**Fig. (5):**
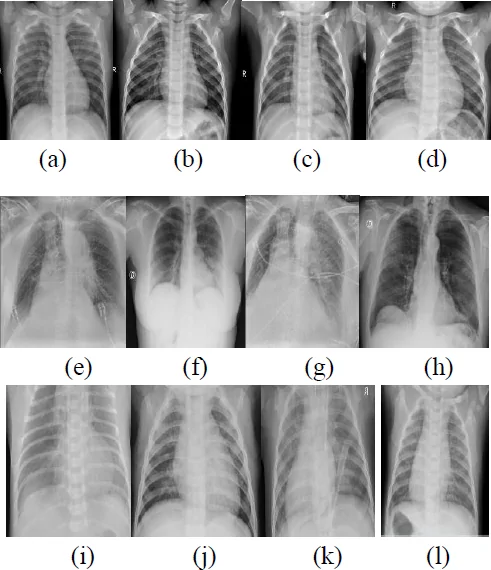
The dataset sample images belong to the different classes: (**a-d**) Normal, (**e-h**) Covid-19, and (**i-l**) Pneumonia.

**Fig. (6):**
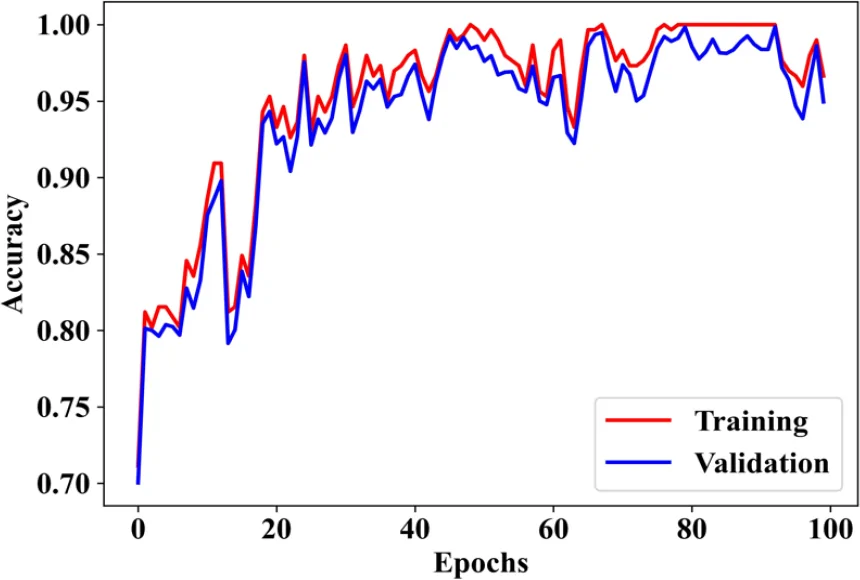
Validation of proposed work with training and validation accuracy.

**Fig. (7):**
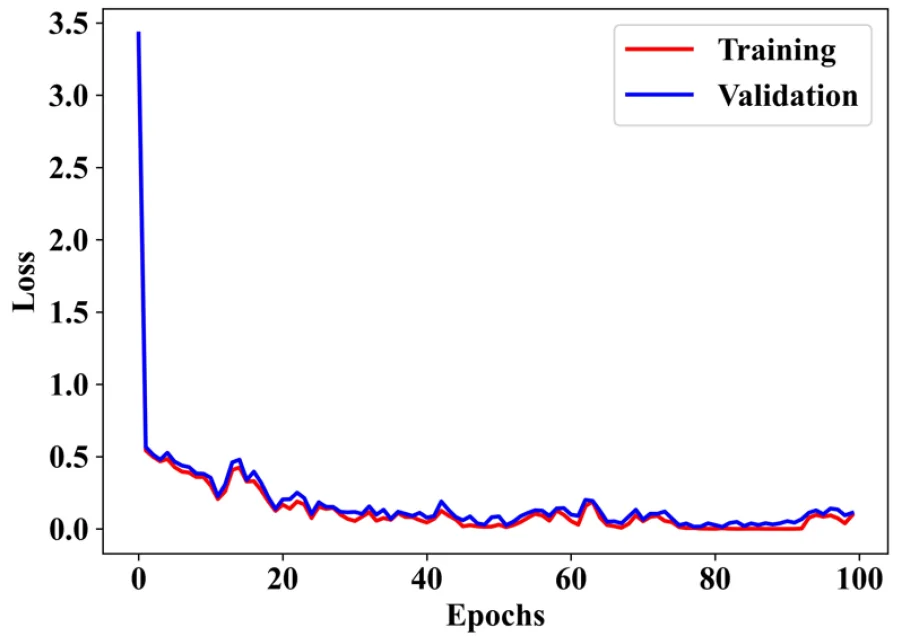
Training and validation loss over epochs.

**Fig. (8):**
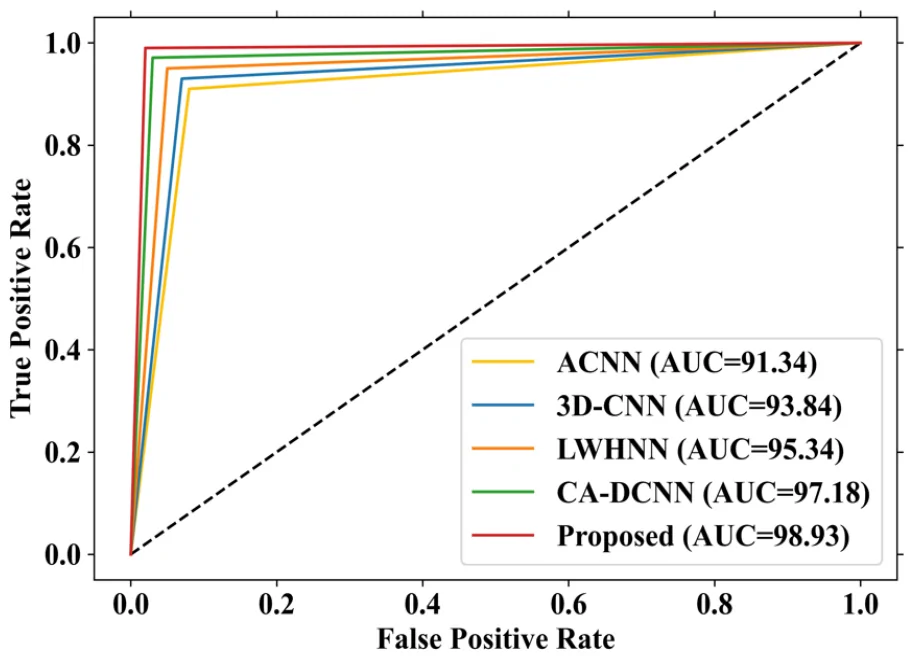
ROC curves and AUC comparison across classes.

**Fig. (9):**
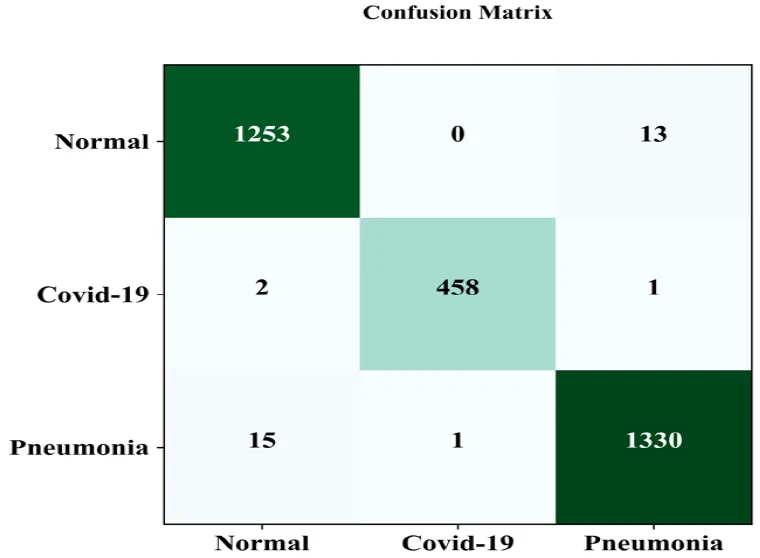
Confusion matrix for the proposed work.

**Fig. (10):**
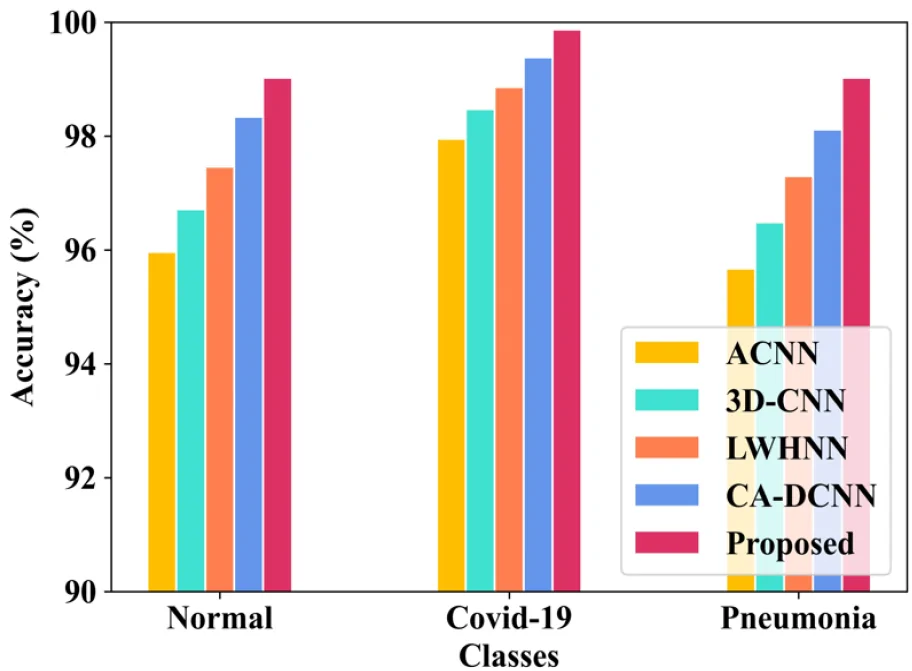
Accuracy *vs*. different classes.

**Fig. (11):**
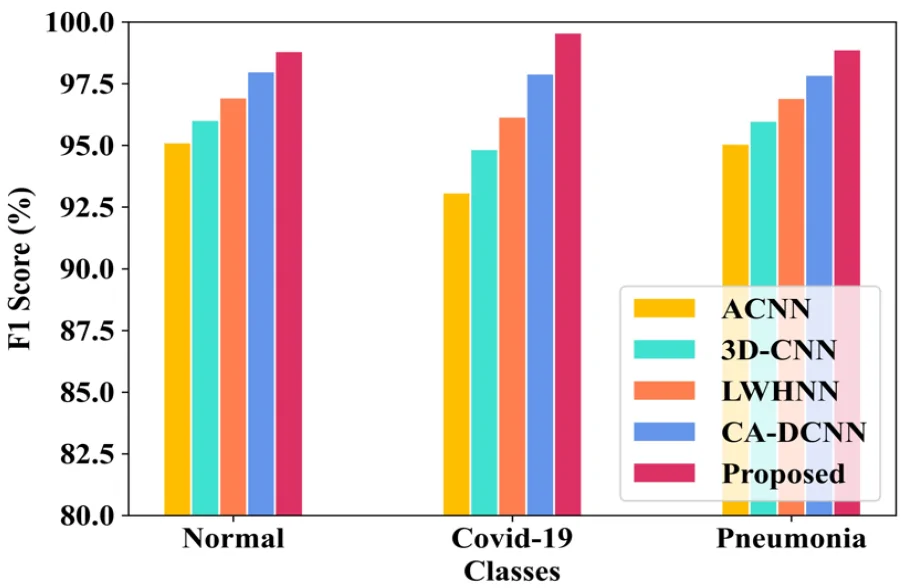
F1-score *vs*. different classes.

**Fig. (12):**
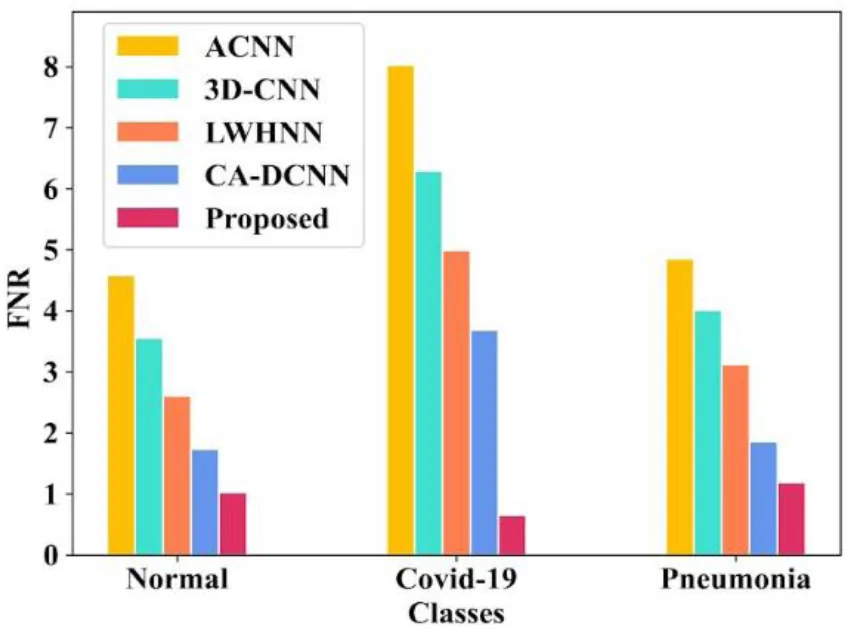
False negative ratio *vs*. different classes.

**Fig. (13):**
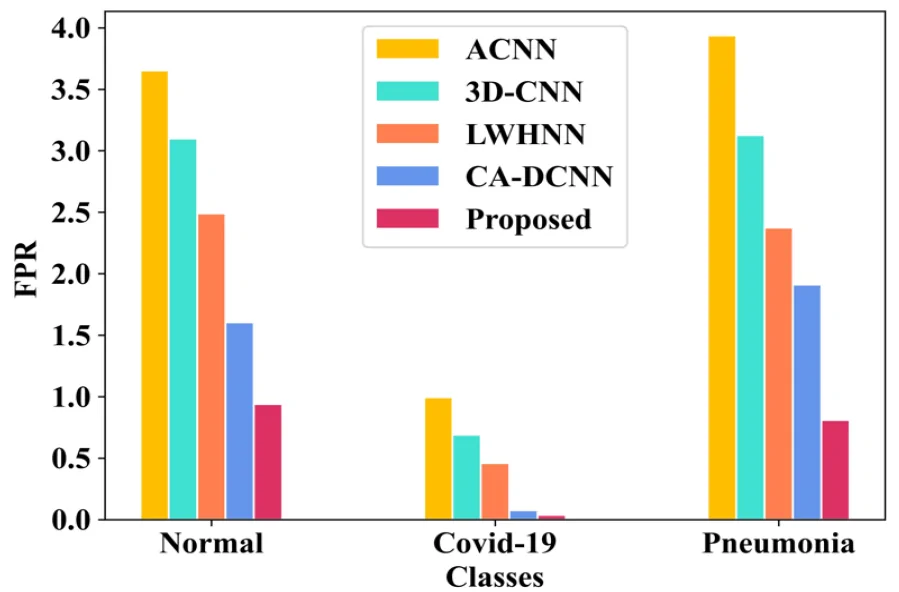
False positive ratio *vs*. different classes.

**Fig. (14):**
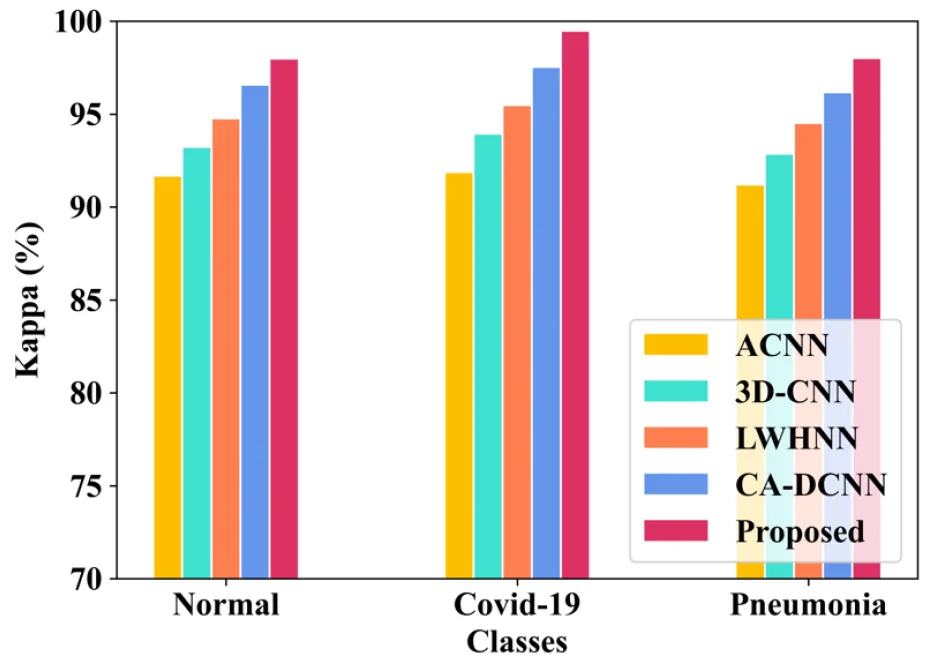
Kappa *vs*. different classes.

**Fig. (15):**
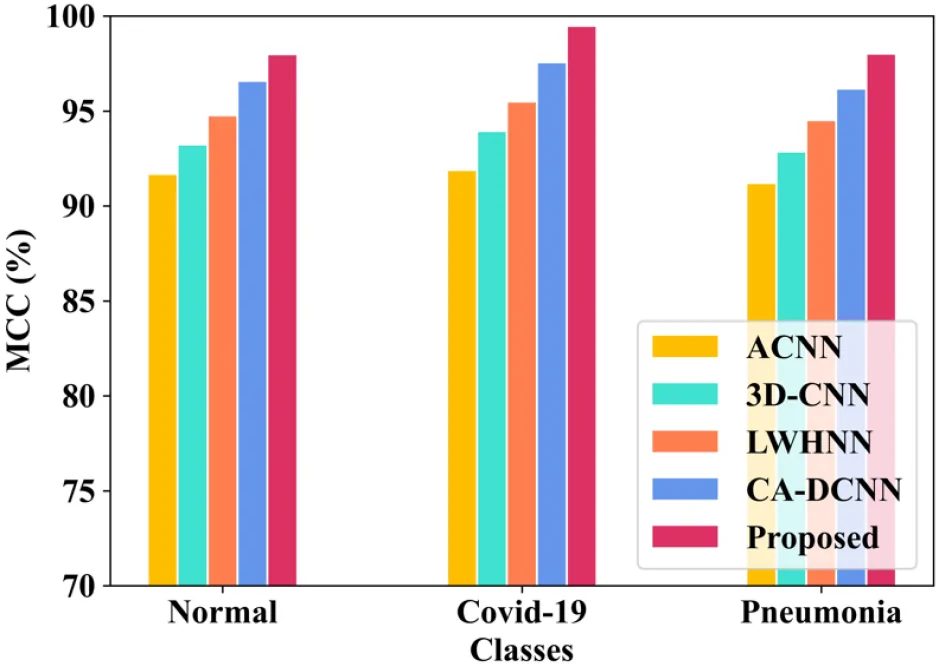
MCC *vs*. different classes.

**Fig. (16):**
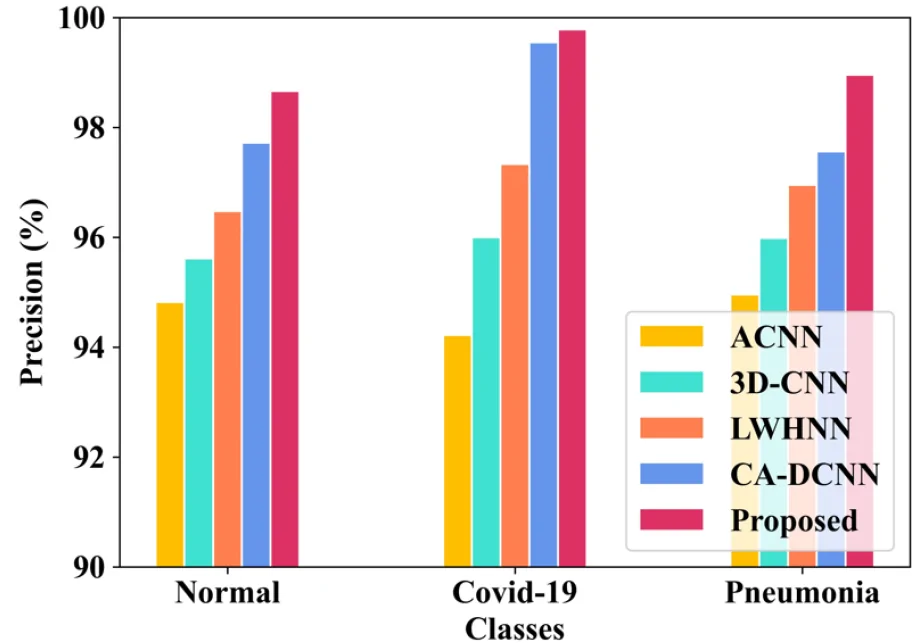
Precision *vs*. different classes.

**Fig. (17):**
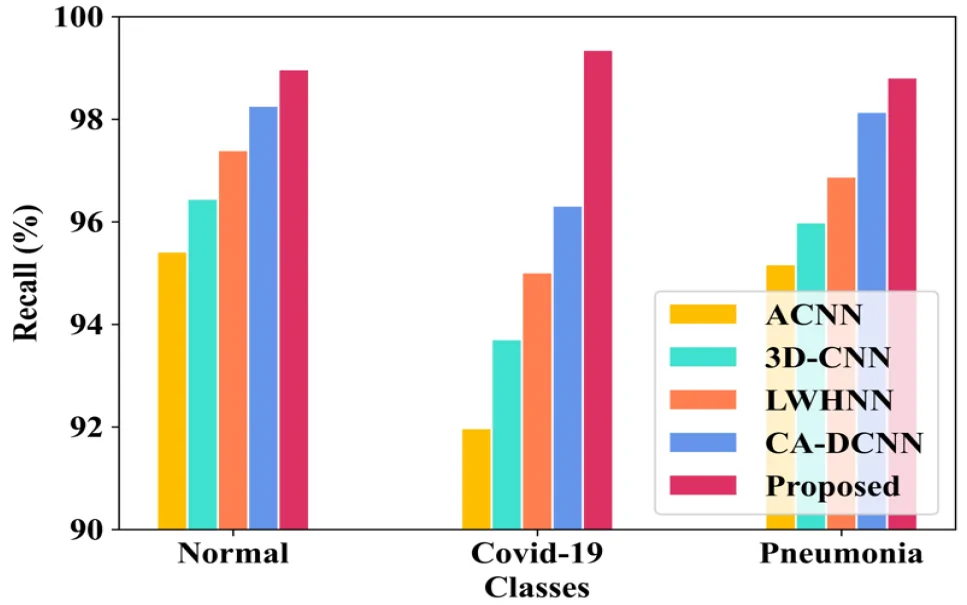
Recall *vs*. different classes.

**Fig. (18):**
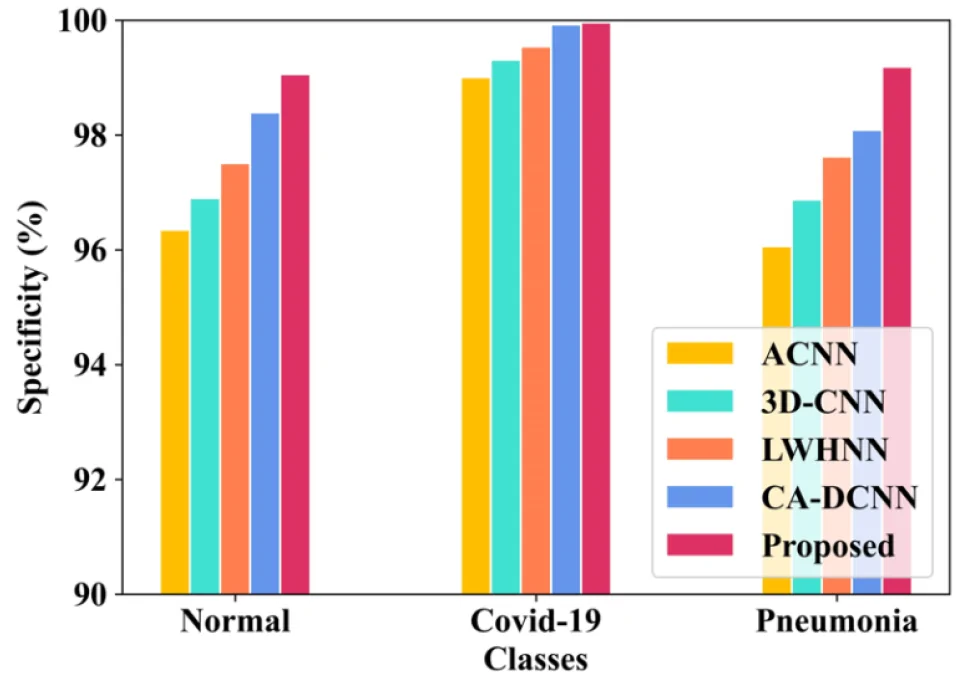
Specificity *vs*. different classes.

**Table 1:** Details based on dataset description.

**Parameters**	**Ranges**
Total X-ray images	6432
No.of images belongs to normal	1,583
No.of images belongs to pneumonia	576
No.of images belongs to Covid-19	4,273
Total number of training samples	5,144
Total number of testing samples	1,288

**Table 2:** Comparative analysis belongs to the execution time.

**Approaches**	**Normal**	**Covid-19**	**Pneumonia**
ACNN [9]	32s	60s	54s
3D-CNN [10]	56s	54s	40s
LWHNN [11]	26s	37s	25s
CA-DCNN [12]	19s	15s	17s
Proposed	2.8s	3s	2.5s

## Data Availability

All data generated or analyzed during this study are included in this published article.

## LIST OF ABBREVIATIONS

ACNN: Attention-based Convolutional Neural Network
**MCSA**: Multi Convolution Spatial Attention
**COVID-19**: Coronavirus disease 2019
**LW-HNN**: Light Weighted Hybrid Neural Network
**MHSAL**: Multi head self attention layer
**GELU**: Gaussian error linear unit
CRKO: Capuchin Red Kite Optimization
**ST-CBAM**: Swin Transformer-based Convolutional Block Attention Module
**CA-DCNN**: Convolutional Autoencoder Deep Convolutional Neural Network

## References

[1] Khan W, Zaki N, Ali L (2021). Intelligent pneumonia identification from chest x-rays: A systematic literature review.. IEEE Access.

[2] Chagas J.V.S.D., de A Rodrigues D., Ivo R.F., Hassan M.M., de Albuquerque V.H.C., Filho P.P.R. (2021). A new approach for the detection of pneumonia in children using CXR images based on an real-time IoT system.. J. Real-Time Image Process..

[3] Mahbub M.K., Biswas M., Gaur L., Alenezi F., Santosh K.C. (2022). Deep features to detect pulmonary abnormalities in chest X-rays due to infectious diseaseX: Covid-19, pneumonia, and tuberculosis.. Inf. Sci..

[4] Zhang J., Xie Y., Pang G., Liao Z., Verjans J., Li W., Sun Z., He J., Li Y., Shen C., Xia Y. (2021). Viral pneumonia screening on chest X-rays using confidence-aware anomaly detection.. IEEE Trans. Med. Imaging.

[5] Qian X., Fu H., Shi W., Chen T., Fu Y., Shan F., Xue X. (2020). M^3^ Lung-Sys: A deep learning system for multi-class lung pneumonia screening from CT imaging.. IEEE J. Biomed. Health Inform..

[6] Ling G., Cao C. (2020). Atomatic detection and diagnosis of severe viral pneumonia CT images based on LDA-SVM.. IEEE Sens. J..

[7] Nahiduzzaman M, Goni MOF, Anower MS, Islam MR, Ahsan M, Haider J, Gurusamy S, Hassan R, Islam MR (2021). A novel method for multivariant pneumonia classification based on hybrid CNN-PCA based feature extraction using extreme learning machine with CXR images.. IEEE Access.

[8] Huang D., Wang L., Wang W. (2023). A multi-center clinical trial for wireless stethoscope-based diagnosis and prognosis of children community-acquired pneumonia.. IEEE Trans. Biomed. Eng..

[9] Feng Y., Yang X., Qiu D., Zhang H., Wei D., Liu J. (2022). Pcxrnet: Pneumonia diagnosis from chest x-ray images using condense attention block and multiconvolution attention block.. IEEE J. Biomed. Health Inform..

[10] Ouyang X., Huo J., Xia L., Shan F., Liu J., Mo Z., Yan F., Ding Z., Yang Q., Song B., Shi F., Yuan H., Wei Y., Cao X., Gao Y., Wu D., Wang Q., Shen D. (2020). Dual-sampling attention network for diagnosis of COVID-19 from community acquired pneumonia.. IEEE Trans. Med. Imaging.

[11] Li Q., Ning J., Yuan J., Xiao L. (2021). A depthwise separable dense convolutional network with convolution block attention module for COVID-19 diagnosis on CT scans.. Comput. Biol. Med..

[12] Asswin C.R. (2023). Transfer learning approach for pediatric pneumonia diagnosis using channel attention deep CNN architectures.. Eng. Appl. Artif. Intell..

[13] Wong P.K., Yan T., Wang H., Chan I.N., Wang J., Li Y., Ren H., Wong C.H. (2022). Automatic detection of multiple types of pneumonia: Open dataset and a multi-scale attention network.. Biomed. Signal Process. Control.

[14] Wang J., Bao Y., Wen Y., Lu H., Luo H., Xiang Y., Li X., Liu C., Qian D. (2020). Prior-attention residual learning for more discriminative COVID-19 screening in CT images.. IEEE Trans. Med. Imaging.

[15] Malik H., Anees T., Din M., Naeem A. (2023). CDC_Net: Multi-classification convolutional neural network model for detection of COVID-19, pneumothorax, pneumonia, lung Cancer, and tuberculosis using chest X-rays.. Multimedia Tools Appl..

[16] Li X., Li L., Jiang Y., Wang H., Qiao X., Feng T., Luo H., Zhao Y. (2025). Vision-language models in medical image analysis: From simple fusion to general large models.. Inf. Fusion.

[17] Liu W., Ni Z., Chen Q., Ni L. (2023). Attention-guided partial domain adaptation for automated pneumonia diagnosis from chest x-ray images.. IEEE J. Biomed. Health Inform..

[18] Ünal Z. Image preprocessing techniques applied on NIR images for fruit bruise detection.. BIO web of conferences.

[19] Athisayamani S., Antonyswamy R.S., Sarveshwaran V., Almeshari M., Alzamil Y., Ravi V. (2023). Feature extraction using a residual deep convolutional neural network (ResNet-152) and optimized feature dimension reduction for MRI brain tumor classification.. Diagnostics.

[20] Dutta P., Sathi K.A., Hossain M.A., Dewan M.A.A. (2023). Conv-ViT: A convolution and vision transformer-based hybrid feature extraction method for retinal disease detection.. J. Imaging.

[21] Tabbakh A, Barpanda SS (2023). A deep features extraction model based on the transfer learning model and vision transformer “tlmvit” for plant disease classification.. IEEE Access.

[22] Liu X., Zhang B., Li X., Liu S., Yue C., Liang S.Y. (2023). An approach for tool wear prediction using customized DenseNet and GRU integrated model based on multi-sensor feature fusion.. J. Intell. Manuf..

[23] Deshmukh P., Satyanarayana G.S.R., Majhi S., Sahoo U.K., Das S.K. (2023). Swin transformer based vehicle detection in undisciplined traffic environment.. Expert Syst. Appl..

[24] Chen B., Zhang Z., Liu N., Tan Y., Liu X., Chen T. (2020). Spatiotemporal convolutional neural network with convolutional block attention module for micro-expression recognition.. Information.

[25] Gong H., Mu T., Li Q., Dai H., Li C., He Z., Wang W., Han F., Tuniyazi A., Li H., Lang X., Li Z., Wang B. (2022). Swin-transformer-enabled YOLOv5 with attention mechanism for small object detection on satellite images.. Remote Sens..

[26] Alruwaili FF, Asiri MM, Alrayes FS, Aljameel SS, Salama AS, Hilal AM (2023). Red kite optimization algorithm with average ensemble model for intrusion detection for secure IoT.. IEEE Access.

[27] Gao L., Chen L., Liu P., Jiang Y., Li Y., Ning J. (2024). Transformer-based visual object tracking *via* fine–coarse concatenated attention and cross concatenated MLP.. Pattern Recognit..

[28] Nagaraju S, Kumar KV, Rani BP, Lydia EL, Ishak MK, Filali I, Karim FK, Mostafa SM (2023). Automated diabetic foot ulcer detection and classification using deep learning.. IEEE Access.

[29] Nalinipriya G., Suneetha M., Mikhailova M., Ramesh S.N., Kumar K.V. (2025). Leveraging double-valued neutrosophic set for real-time chronic kidney disease detection and classification.. Int. J. Neutrosophic Sci..

[30] Malleswari PN, Hanuman CRS, Kammampati VR, Swathi S, Raju BE (2024). Deep learning based DWT-Bi-LSTM classifier for enhanced cardiovascular arrhythmia classification.. FOREX Publ.

[31] Mathesul S., Swain D., Satapathy S.K., Rambhad A., Acharya B., Gerogiannis V.C., Kanavos A. (2023). COVID-19 detection from chest X-ray images based on deep learning techniques.. Algorithms.

